# The Oncogenic and Tumor Suppressive Long Non-Coding RNA–microRNA–Messenger RNA Regulatory Axes Identified by Analyzing Multiple Platform Omics Data from Cr(VI)-Transformed Cells and Their Implications in Lung Cancer

**DOI:** 10.3390/biomedicines10102334

**Published:** 2022-09-20

**Authors:** Osama Sweef, Chengfeng Yang, Zhishan Wang

**Affiliations:** Division of Cancer Biology, Department of Medicine, MetroHealth Medical Center, Case Western Reserve University School of Medicine, Cleveland, OH 44109, USA

**Keywords:** hexavalent chromium (Cr(VI)), lncRNA, miRNA, ceRNA, lung cancer, cancer stemness, ROC curve, prognosis

## Abstract

Chronic exposure to hexavalent chromium (Cr(VI)) causes lung cancer in humans, however, the underlying mechanism has not been well understood. Long non-coding RNAs (lncRNAs) and microRNAs (miRNAs) are commonly studied non-coding RNAs. miRNAs function mainly through interaction with the 3′-untranslated regions of messenger RNAs (mRNAs) to down-regulate gene expression. LncRNAs have been shown to function as competing endogenous RNAs (ceRNAs) to sponge miRNAs and regulate gene expression. It is now well accepted that lncRNAs and miRNAs could function as oncogenes or tumor suppressors. Dysregulations of lncRNAs and miRNAs have been shown to play important roles in cancer initiation, progression, and prognosis. To explore the mechanism of Cr(VI) lung carcinogenesis, we performed lncRNA, mRNA, and miRNA microarray analysis using total RNAs from our previously established chronic Cr(VI) exposure malignantly transformed and passage-matched control human bronchial epithelial BEAS-2B cells. Based on the differentially expressed lncRNAs, miRNAs, and mRNAs between the control (BEAS-2B-Control) and Cr(VI)-transformed (BEAS-Cr(VI)) cells and by using the lncRNA–miRNA interaction and miRNA target prediction algorithms, we identified three oncogenic (HOTAIRM1/miR-182-5p/ERO1A, GOLGA8B/miR-30d-5p/RUNX2, and PDCD6IPP2/miR-23a-3p/HOXA1) and three tumor suppressive (ANXA2P1/miR-20b-5p/FAM241A (C4orf32), MIR99AHG/miR-218-5p/GPM6A, and SH3RF3-AS1/miR-34a-5p/HECW2) lncRNA–miRNA–mRNA regulatory axes. Moreover, the relevance of these three oncogenic and three tumor suppressive lncRNA–miRNA–mRNA regulatory axes in lung cancer was explored by analyzing publicly available human lung cancer omics datasets. It was found that the identified three oncogenic lncRNA–miRNA–mRNA regulatory axes (HOTAIRM1/miR-182-5p/ERO1A, GOLGA8B/miR-30d-5p/RUNX2, and PDCD6IPP2/miR-23a-3p/HOXA1) and the three tumor suppressive lncRNA–miRNA–mRNA regulatory axes (ANXA2P1/miR-20b-5p/FAM241A (C4orf32), MIR99AHG/miR-218-5p/GPM6A, and SH3RF3-AS1/miR-34a-5p/HECW2) have significant diagnostic and prognosis prediction values in human lung cancer. In addition, our recent studies showed that Cr(VI)-transformed cells display cancer stem cell (CSC)-like properties. Further bioinformatics analysis identified the oncogenic lncRNA–miRNA–mRNA regulatory axes as the potential regulators of cancer stemness. In summary, our comprehensive analysis of multiple platform omics datasets obtained from Cr(VI)-transformed human bronchial epithelial cells identified several oncogenic and tumor suppressive lncRNA–miRNA–mRNA regulatory axes, which may play important roles in Cr(VI) carcinogenesis and lung cancer in general.

## 1. Introduction

Chromium (Cr) is a naturally occurring heavy metal element and mainly exists in three valences: Cr(0), Cr(III), and Cr(VI). Hexavalent chromium (Cr(VI)) has been classified as a potent Group I carcinogen and exposure to Cr(VI) causes lung cancer in humans [1]. Due to the wide application of Cr(VI) in many industrial manufacturing processes, a significant amount of Cr(VI) has been released into the environment and human exposure to Cr(VI) is common. In fact, Cr(VI) is listed as one of top 20 hazardous substances in waste sites on the U.S. National Priority List [2], which represents a significant environmental health issue. Although the carcinogenicity of Cr(VI) is well recognized, the mechanism of Cr(VI) carcinogenesis has not been clearly defined. Studies showed that Cr(VI) exposure causes genetic and epigenetic dysregulations, which may play important roles in Cr(VI) carcinogenesis [3,4,5,6]. Non-coding RNAs (ncRNAs) refer to RNA molecules that do not have significant protein coding capacities. Regulation of gene expression by ncRNAs is considered an important epigenetic regulatory mechanism of gene expression. However, few studies have been carried out to investigate the effect of chronic Cr(VI) exposure on ncRNAs and the role of ncRNAs in Cr(VI) carcinogenesis is not well understood [7,8].

MicroRNAs (miRNAs) and long non-coding RNAs (lncRNAs) are among the most studied ncRNAs and are critically involved in cancer development and progression by deregulating oncogenic and tumor suppressive gene expressions [9,10]. By interacting with the 3′-untranslated regions of messenger RNAs (mRNAs), miRNAs down-regulate gene expressions at the posttranscriptional level in most cases. In contrast, lncRNAs may up- or down-regulate gene expressions at transcriptional and posttranscriptional levels [10]. LncRNAs can function as competing endogenous RNAs (ceRNAs) sponging miRNAs and forming lncRNA–miRNA–mRNA regulatory axes through RNA–RNA interactions to regulate gene expression [11]. While some studies investigated the effect of Cr(VI) exposure on expressions of individual miRNAs or lncRNAs [7,8], no studies have been carried out to determine the effect of Cr(VI) exposure on RNA–RNA interactions, especially the lncRNA–miRNA–mRNA regulatory axis and its implication in Cr(VI) carcinogenesis.

The three components of the regulatory axis, lncRNA/miRNA/mRNA, form a ceRNA module, having a three-fold enrichment via the participation of each collaborator to accomplish important biological function [12]. Many studies reported that the triple components of lncRNA/miRNA/mRNA serve as critical regulatory axes in many biological processes and human diseases [13]. The action of the ceRNA module is affected by a variety of parameters, including the number of ceRNA components as well as their subcellular localization, the binding affinity of miRNAs to the sponges that they bind to, and RNA secondary structures, RNA editing, and RNA-binding proteins [14]. ceRNA networks can be disrupted by changes in these variables, resulting in human diseases such as cancer [15]. The components of the oncogenic lncRNA/miRNA/mRNA ceRNA module are defined as follows: up-regulated oncogenic lncRNAs, down-regulated tumor suppressive miRNAs, and up-regulated oncogenic mRNAs. In contrast, the tumor suppressive lncRNA/miRNA/mRNA ceRNA module’s components are defined as down-regulated tumor suppressive lncRNAs, up-regulated oncogenic miRNAs, and down-regulated tumor suppressive mRNAs [16].

To investigate the mechanism of Cr(VI) lung carcinogenesis, we performed lncRNA, mRNA, and miRNA microarray analyses using total RNAs from our previously established chronic Cr(VI) exposure malignantly transformed and passage-matched control human bronchial epithelial BEAS-2B cells. We carried out comprehensive bioinformatics analysis of these multi-omics datasets and used lncRNA–miRNA interaction and miRNA target prediction algorithms to identify the oncogenic and tumor suppressive lncRNA–miRNA–mRNA regulatory axes that may play important roles in Cr(VI) carcinogenesis. Moreover, the relevance of the identified oncogenic and tumor suppressive axes in lung cancer in general was also explored by analyzing publicly available human lung cancer multi-omics datasets.

## 2. Material and Methods

### 2.1. Cell Culture and Microarray Analyses

The establishment and characterization of immortalized human bronchial epithelial BEAS-2B cell transformation by chronic Cr(VI) exposure were reported in our recent publication [17]. The passage-matched control (BEAS-2B-Control) and chronic Cr(VI) exposure-transformed BEAS-2B cells (BEAS-2B-Cr(VI)) were cultured in Dulbecco’s modified Eagle medium (DMEM) (Thermo Fisher, MA) supplemented with 1% penicillin–streptomycin (P/S) and 5% fetal bovine serum (FBS). Total RNAs were purified from BEAS-2B-Control and BEAS-2B-Cr(VI) cells according to the instructions of the TRIzol™ reagent (Life Technologies, Carlsbad, CA, USA). RNA samples were shipped to ArrayStar Inc (Rockville, MD, USA) and LC Sciences, LLC (Houston, TX, USA) for lncRNA, mRNA, and miRNA microarray analyses, respectively.

### 2.2. Gene Set Enrichment (GSE) Analysis

Differentially expressed (DE) genes of lncRNA, miRNA, and mRNA of each array profile between BEAS-2B-Control and BEAS-2B-Cr(VI) cells were analyzed. Hierarchical clustering was performed using HeatMapper to represent the relationships between the samples based upon the expression of a transcripts per million (TPM) data matrix of each expression profile for lncRNA, miRNA, and mRNA. The GSE analyzed the profile of DE lncRNAs, miRNA, and mRNA and derived their biological information from the source, gene ontology (GO), biological pathways, miRNA target, and human protein databases. GO terms are molecular function (MF), cellular components (CC), and biological processes (BP). GSE analyses implemented with Gprofiler (https://biit.cs.ut.ee/gprofiler (accessed on 10 November 2021)) for lncRNAs and mRNAs and ToppGene (https://toppgene.cchmc.org/ (accessed on 10 November 2021)) for miRNA functional analysis with a *p*-value less than 0.05 were considered significantly enriched by DE candidates.

### 2.3. Construction of the lncRNA/miRNA/mRNA Regulatory ceRNA Module Network

Based on the ceRNA hypothesis, lncRNA acts as an endogenous “sponge” to sink miRNA and regulate mRNA expression. There are miRNAs and lncRNAs or mRNAs with inverse expression levels in ceRNA scaffold in an oncogenic regulatory module which is up-regulated lncRNA/down-regulated miRNA/up-regulated mRNA and in a tumor suppressor regulatory module which is down-regulated lncRNA/up-regulated miRNA/down-regulated mRNA. The Gprofiler platform was first used to predict and select miRNAs that could interact with up- and down-regulated lncRNAs and mRNAs in the array profiles of Cr(VI)-transformed cells. The predicted miRNAs were then matched with actually differentially expressed miRNAs in the miRNA microarray profile of Cr(VI)-transformed cells. This matching identified 10 oncogenic and 13 tumor suppressor miRNAs, which were used to build oncogenic and tumor suppressive lncRNA/miRNA/mRNA regulatory axes, respectively. More specifically, each selected miRNA was matched with predicted interacting oncogenic or tumor suppressive lncRNA and mRNA partners that were differentially expressed (DE) in Cr(VI)-transformed cells. Finally, three oncogenic and tumor suppressive lncRNA/miRNA/mRNA regulatory axes were selected based on the following criteria: (1) the relevance to cancer; (2) *p*-value for the significance of differential expression in the array profiles of Cr(VI)-transformed cells; (3) fold changes of the lncRNA in each axis ≥ 2.0. The DE lncRNA–miRNA and DE mRNA–miRNA targeting was predicted using the miRtarBase, TargetScan, and StarBase databases. The ceRNA networks were constructed and visualized via the Cytoscape platform (https://cytoscape.org/ (accessed on 22 December 2021)).

### 2.4. Cancer Stemness Signature Analysis

The cancer stemness molecular signature is able to classify lung cancer patients into high- and low-risk clusters based on the regulatory and physical interaction of stemness-related genes or the stemness transcription factors (TFs) with ceRNA elements. The stemness signature was tested via the Stem Cell Network (SCN) platform (http://stemcellnet.sysbiolab.eu (accessed on 5 May 2022)), and ceRNA input genes were identified as a potential target genes in ChIP-chip and ChIP-Seq studies of pluripotency-associated TFs in the stem cell dataset. The SCN investigates the physical and regulatory interactions of ceRNA component candidates with stemness TFs and in the stemness signature dataset. The modules of ceRNAs were also displayed as an interactive network, revealing the stemness characteristic of each ceRNA’s components.

### 2.5. The ceRNA Modules and Survival Analysis Using TCGA Datasets

High-throughput experimental data with survival profiles of lung cancer patients were extracted from the clinical data of The Cancer Genome Atlas (TCGA) of lung adenocarcinoma (LUAD) and lung squamous cell carcinoma (LUSC) sources. TCGA_LUAD and TCGA_LUSC project datasets were used to analyze the expression levels of the ceRNA module components. For survival analysis, the data were obtained by the Cbioportal platform (https://www.cbioportal.org/datasets (accessed on 28 January 2022)). The sequencing profiles of LUAD and LUSC cases (TCGA, Cell 2018) were used from the TCGA, PanCancer Atlas. The survival analysis package in MedCalc (https://www.medcalc.org/ (accessed on 25 May 2022)) was used to correlate between prognosis of the ceRNA collaborators and the overall survival of lung cancer patients. The relative risk score was estimated by hazard ratio (HR) between high- and low-risk groups. The *p*-values less than 0.05 recognized the survival rate of lung cancer patients as statistically significant.

### 2.6. The Receiver Operating Characteristic (ROC) Curve Analysis

An excellent diagnostic test evaluation method is the receiver operating characteristic (ROC) curve which is a depiction of test sensitivity vs. 100-specificity or false positive rate (FPR) with the x coordinate as the reference point. The ROC curve analysis was utilized to determine the potential diagnostic significance of the identified ceRNA modules in the context of lung cancer. The calculation of the ROC and the area under the ROC curve (AUC) was accomplished with the help of the ROC package. The area under curve (AUC) values of 0.7:0.8, 0.8:0.9, or over 0.9 are indications of a good, outstanding, or unique biomarker, respectively. The ROC analysis package in the R program (https://www.rstudio.com/ (accessed on 25 May 2022)) was used to calculate the AUC of each ceRNA module’s components. The log-rank test was used to examine the biomarkers in the ceRNAs and the ROC curves of lung cancer patients. The *p*-values of less than 0.05 were considered to be statistically significant, indicating a high degree of diagnostic accuracy, sensitivity, and specificity for the biomarkers.

## 3. Results

### 3.1. Microarray Gene Expression Profiling and Identification of DE of lncRNAs, miRNAs, and mRNAs

**Figure 1:**
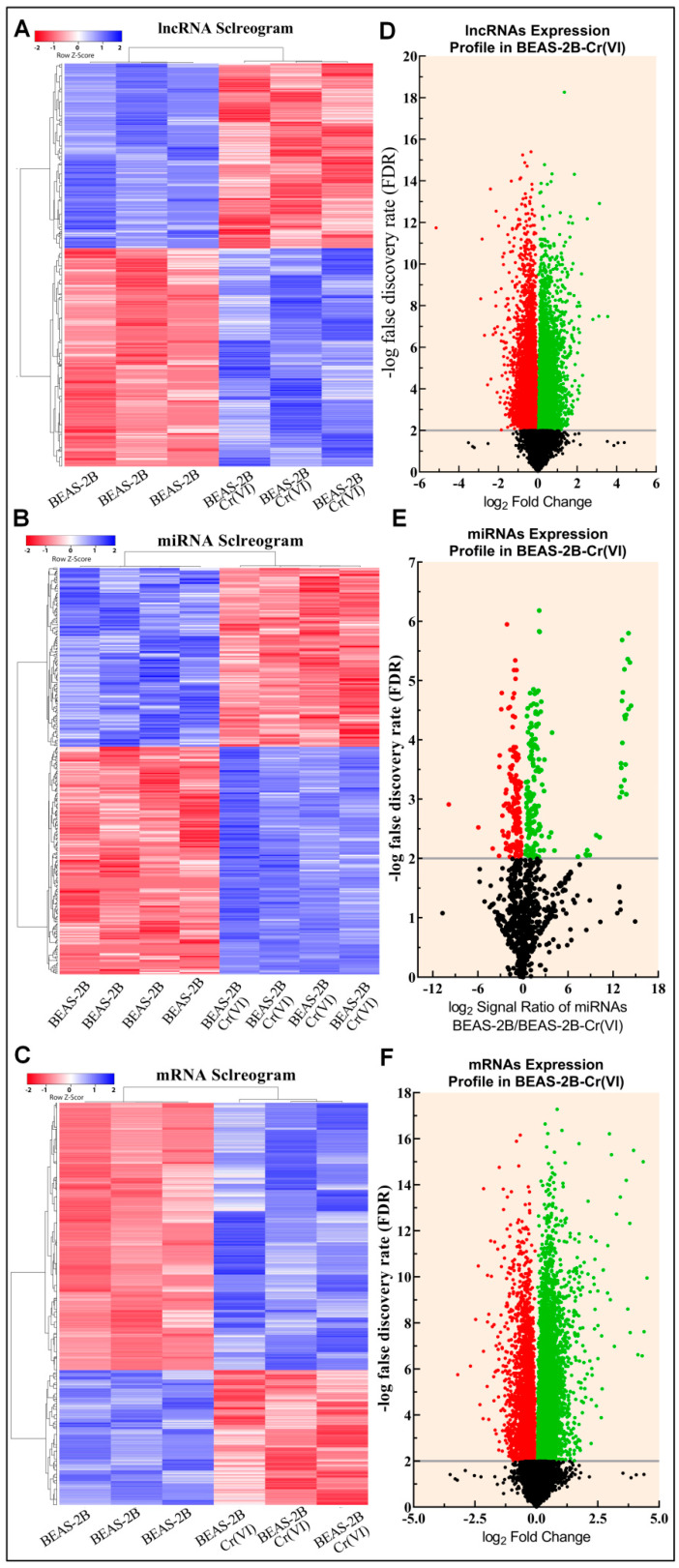
Transcriptomic profiles of BEAS-2B-Control and BEAS-2B-Cr(VI) cells. (**A**–**C**) Hierarchical clustering correlogram of DE lncRNA, miRNA, and mRNA transcripts, respectively. In the two-color system, blue indicates increased expression, while red indicates decreased expression. (**D**–**F**) Volcano graphs show the DE RNA transcripts of the lncRNA, miRNA, and mRNA transcripts, respectively. The significantly up-regulated candidates are marked by green dots; the significantly down-regulated candidates are marked by red dots whereas the non-significant candidates are marked by black dots under the gray line.

With the help of microarray analysis, we were able to collect the expression profiles of lncRNAs, miRNAs, and mRNAs from passage-matched control (BEAS-2B-Control) cells and Cr(VI)-transformed cells (BEAS-2B-Cr(VI)). The expansion levels are shown in a clustered heatmap using blue–red colors to display separate DE candidates in each profile of interest. In Figure 1A–C, the correlograms of lncRNA, miRNA, and mRNA are depicted unbiasedly. The statistical significance of the minus log false discovery rate (−log FDR) is shown against the extent of change in expression levels (log_2_ fold change) of individual transcripts in a volcano plot. The volcano plots show the differential expression of lnRNAs, miRNAs, and mRNAs (# *p*-value ≤ 0.05 or # −log FDR value cut-off: ≥2.0). There were 4559 lncRNAs that showed differential expression after Cr(VI)-induced cell malignant transformation. The expression of 2602 lncRNAs was increased, while the expression of 1957 lncRNAs was decreased (Figure 1D, Supplementary Excel File S1). Similarly, 1072 miRNAs were differentially expressed, 565 of which were up-regulated and 507 were down-regulated (Figure 1E, Supplementary Excel File S2). Additionally, 4300 mRNAs were found to be differently expressed, with 2876 of them being up-regulated and 1424 being down-regulated (Figure 1F, Supplementary Excel File S3).

### 3.2. Functional Annotation Analysis of DE lncRNA, miRNA, and mRNA

**Figure 2:**
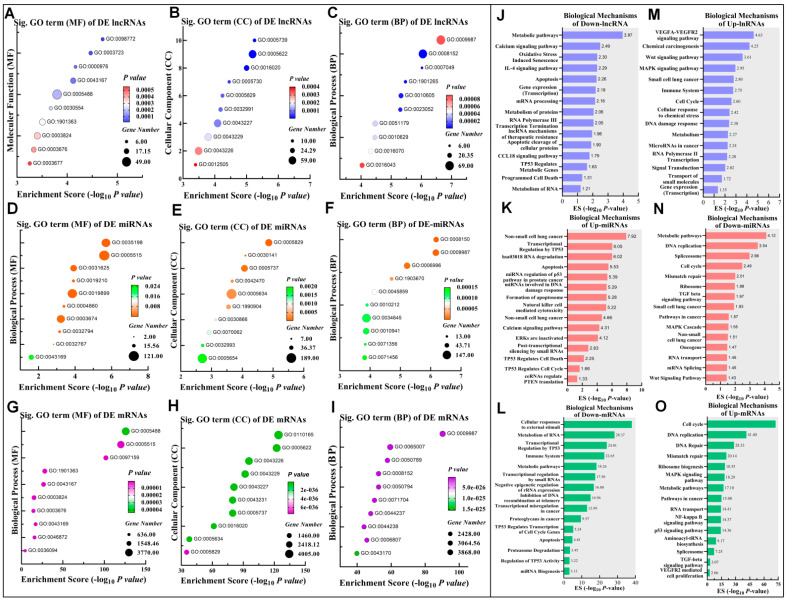
Biological profiles of DE lncRNA, miRNA, and mRNA transcripts. (**A**) Top significant GO terms of linked MF DE lncRNA profile. (**B**) Top significant CC. (**C**) Top significant BP of DE lncRNA profile. (**D**–**F**) Top significant MF, CC, and BP from the DE miRNAs, respectively. The top significant terms of MF, CC, and BP from the DE mRNAs are represented in (**G**–**I**), respectively. The top significant biological pathways of down-lncRNA, up-miRNA, and down-mRNA (tumor suppressor axis) are shown in (**J**–**L**). The significant biological pathways of the oncogenic axis of Up-lncRNA, Down-miRNA, and Up-mRNA are represented in (**M**–**O**). The dot size reflects the number of shared genes in each biological function and the dot two-color system indicates the *p*-values.

The analyses of gene ontology (GO) enrichment and biological pathways were carried out using TopGO, Gprofiler, and miEAA in order to shed light on the roles of DE lncRNA, miRNA, and mRNAs in Cr(VI)-transformed BEAS-2B cells (# fold change cut-off: ≥±1.0 and # *p*-value cut-off: ≤0.05). The DE lncRNAs, miRNAs, and mRNAs are involved in biological processes (BP), cellular components (CC), and molecular functions (MF) as defined by the GO terms. The top 10 enriched GO terms of MF, BP, and CC were identified for each RNA profile (Figure 2A–I). Moreover, cell cycle, metabolic process, signaling, RNA binding cytosolic, nucleus, and more were identified as significantly enriched GO terms (Supplementary Tables S1–S3).

The DE RNAs are categorized into up-lncRNA/down-miRNA/up-mRNA as an oncogenic axis and down-lncRNA/up-miRNA/down-mRNA as a tumor suppressor regulatory axis. The biological mechanisms were enriched based on the Kyoto Encyclopedia of Genes and Genomes (KEGG), Reactome (REAC), and Wiki (WP) pathways. The most enriched pathways to the tumor suppressor axis were linked mainly with apoptosis, TP53 activity, transcriptional misregulation in cancer, and negative regulation of the MAPK pathway (Figure 2J–L). On the other hand, cell cycle, pathways in cancer, ribosome biogenesis mRNA splicing, and MAPK signaling pathway were among the pathways most often related to the oncogenic axis (Figure 2M–O).

### 3.3. Oncogenic and Tumor Suppressive ceRNA Module–Protein Interactions

**Figure 3:**
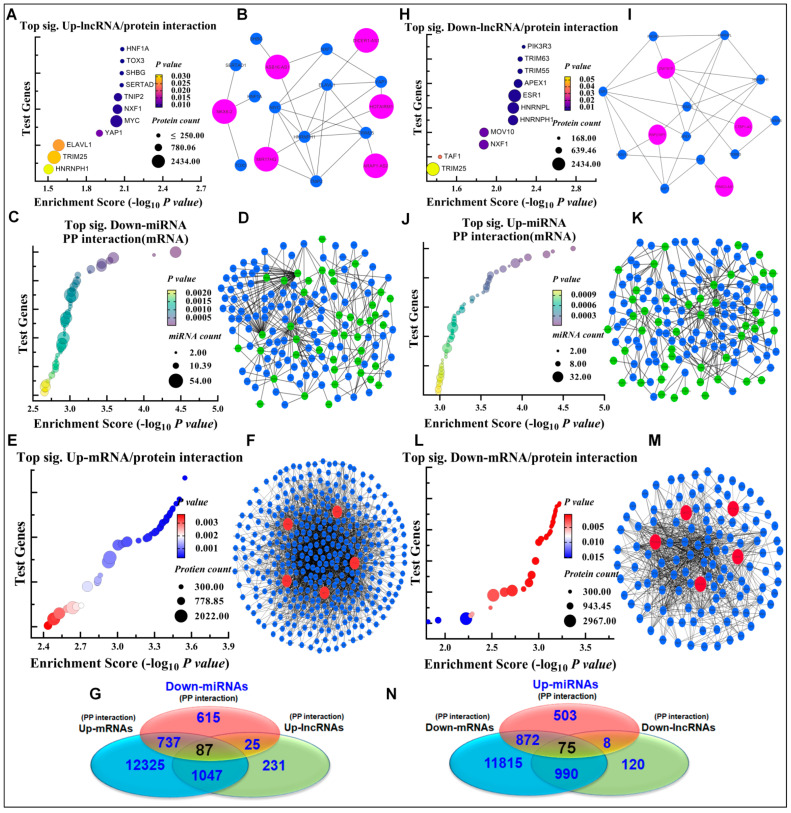
RNA–protein binding networks of oncogenic and tumor suppressive axes. (**A**) Top up-lncRNAs/protein–protein (PP) interaction, demonstrating considerable one seed interaction through the examined tested genes. (**B**) The top up-lncRNAs and PP interaction network, which illustrates all the roles of up-lncRNAs and PP interactions as network panels. (**C**) Top 50 down-miRNAs that bind to mRNA. (**D**) Connections between down-miRNAs and mRNA in the top 50 partners. (**E**) The top 5 up-mRNAs interacting with PP interactions (one seed). (**F**) A connection between the PP network (one seed) and the top five down-mRNAs. (**G**) Venn diagram illustrating the shared proteins interacting with the oncogenic axis components, 87 proteins are interacting with the oncogenic axis components. (**H**) The linkage between top down-regulated lncRNAs and PP interaction (one seed). (**I**) The network panel of PP interaction with the top down-regulated lncRNAs. (**J**) The highly significant top 50 interactions of up-miRNAs with their partners (mRNA). (**K**): Up-miRNA–PP interaction network (mRNA) (top 50 interactions). (**L**) The highly significant top 5 interactions of down-mRNAs with their protein partners. (**M**) The network connections between the PP interactions and the top 5 down-mRNAs. (**N**) Venn diagram depicting common proteins interacting with the tumor suppressor system components, 75 proteins are involved. The dot size reflects the interactant counts of proteins and the dot two-color system indicates the *p*-value significance of RNA/PP interaction. The lncRNAs, miRNAs, mRNAs and proteins are labeled in violet, green, red, and blue colors, respectively, in the RNA–protein binding network.

The RNA–protein connections in Cr(VI)-transformed BEAS-2B cells were identified by the prioritization of the genes in the ToppGene database that share the functional similarities with the “seed” list. High-confidence RNA–protein interactions (one seed) from the ToppGene database were used in this investigation to identify proteins that have a key role in tumor suppressive and oncogenic regulatory axes. Tumor suppressive and oncogenic axes of DE transcripts (lncRNA, miRNA, and mRNA) were recruited to identify their own protein-binding partners. The oncogenic partners comprise three highly significant binding components: NKX6-2lncRNA/HNF1A, miR-491-3p/ZNF226, and TCF4/WASF4P (Figure 3A–F; Supplementary Excel Files S4 and S5). The tumor suppressive partners comprise three highly significant binding components: MIR99AHGlncRNA/PIK3R3, miR-661/ZYG11A, and PPP2R2B/ZNF136 (Figure 3H–M; Supplementary Excel Files S4 and S5). More precisely, along the oncogenic triple axis, 1390 and 14,195 proteins were found to interact with 4214 up-lncRNAs and 2875 up-mRNAs, respectively; and 87 proteins were common to all three components of the oncogenic triple axis (Figure 3G; Supplementary Excel Files S4 and S6). In addition, the tumor suppressive partners contain 4078 down-lncRNAs and 1423 down -mRNAs, each of which interacts with 1003, and 13,752 proteins, correspondingly. The tumor suppressive triple axes shared 75 proteins in common among their three components (Figure 3N; Supplementary Excel Files S4 and S6).

### 3.4. Identification of Oncogenic and Tumor Suppressor ceRNA Modules

**Figure 4:**
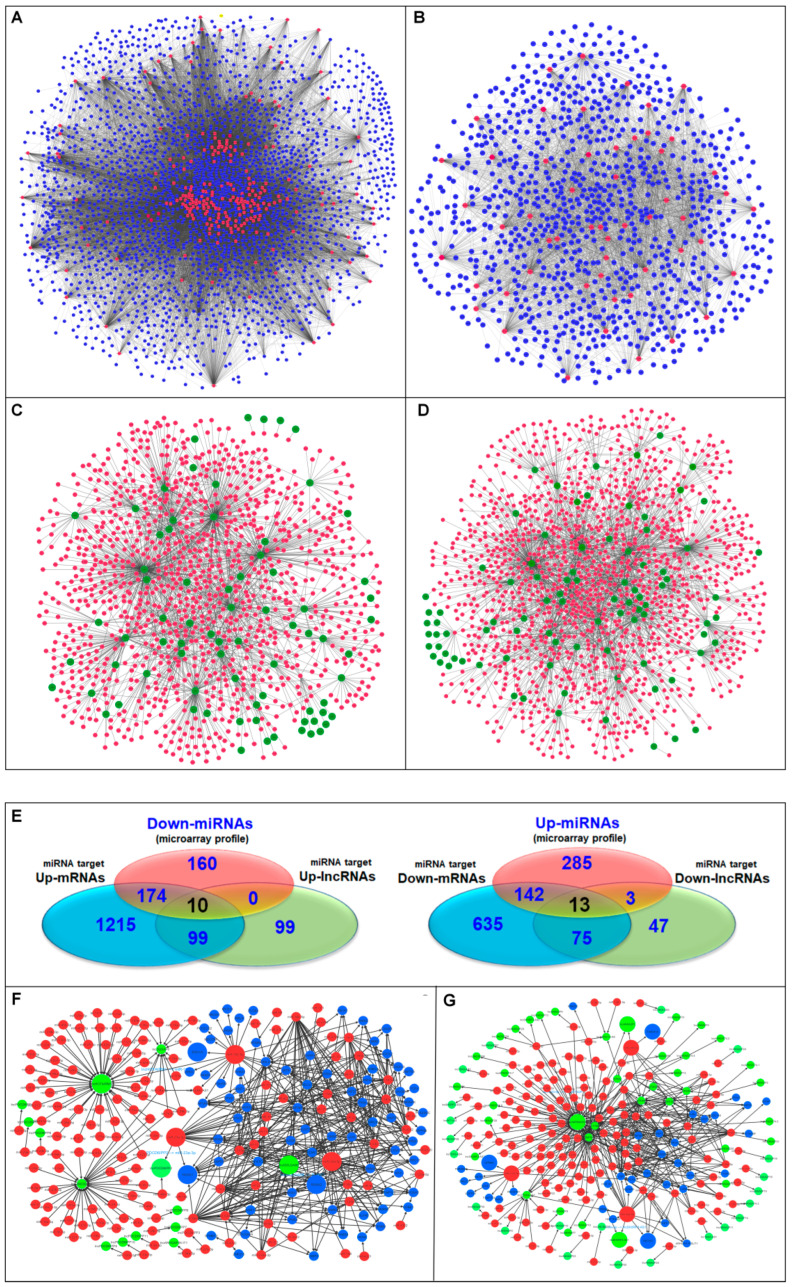
miRNA interactions with lncRNA and mRNA of BEAS-2B-Cr(VI). The miRNA interactions with mRNAs that have been up- and down-regulated are shown in (**A**,**B**). miRNA interactions with lncRNAs that have been up- and down-regulated are shown in (**C**,**D**), respectively. (**E**) The shared miRNAs in array profile and predictably interacting with DE lncRNAs and mRNAs in oncogenic and tumor suppressor activities. Triple networks comprising lncRNA/miRNA/mRNA in oncogenic and tumor suppressor activities are shown in (**F**,**G**), respectively. The three-color system of green, red, and blue represents lncRNA, miRNA, and mRNA, respectively. Gene ID of mRNA and lncRNA (last 6 digits only) was used in each node of the network.

**Figure 5:**
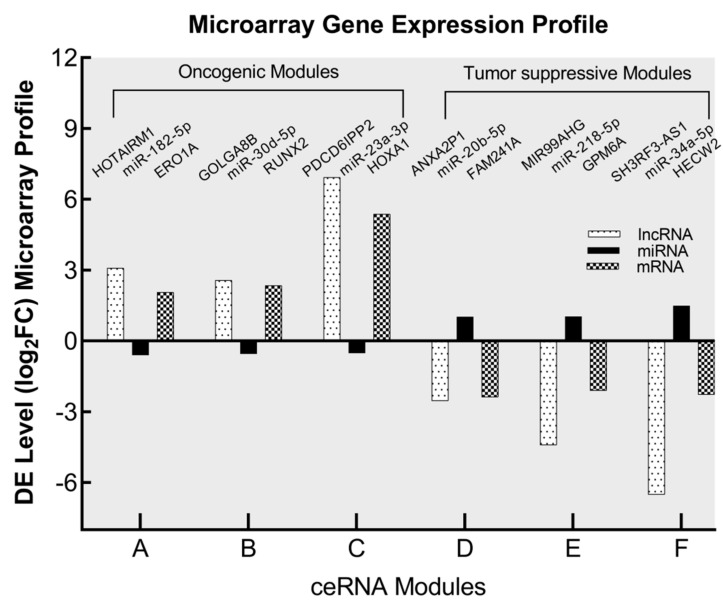
The potential oncogenic and tumor suppressor RNAs in BEAS-2B-Cr(VI) cells. The fold changes (compared to control cells) of expression levels of each RNA component are calculated using values from each type of RNA microarray expression profile.

Signal transduction is affected by the binding of the miRNA/mRNA axis to lncRNA, playing an important role in the onset and progression of cancer. We conducted this lncRNA-associated ceRNA analysis based on a hypothesis of ceRNA of up- or down-regulation of each lncRNA, miRNA, and mRNA. According to the results of the targeting analysis, there are 1692 miRNAs that target 297 DE lncRNAs, whereas 2571 miRNAs target 3508 DE mRNAs (Figure 4A,B; Supplementary Excel Files S4 and S6). The miRNA array data profile was utilized as well for the sake of achieving accuracy and reliability. When the DE miRNA candidates from the miRNA array profile were matched to the anticipated miRNA targets of lncRNA and mRNA, 10 candidates were revealed in the down-miRNA profile and these candidates were expected to interact with both up-lncRNA and up-mRNA (FC ≥ 2) at the same time (Figure 4C,E). The regulatory axis for oncogenic activity is provided by the up-lncRNA/down-miRNA/up-mRNA components. On the other hand, the miRNA array profile showed that 13 candidates were up-regulated, and these candidates were anticipated to interact with down-lncRNA as well as down-mRNA (FC ≥ 2) (Figure 4D,E). The components of down-lncRNA/up-miRNA/down-mRNA all work together to operate as a tumor suppressor regulatory axis. The oncogenic activity was determined by screening out the oncogenic regulatory components of miRNAs that interact with up-regulated lncRNAs and up-regulated mRNAs. The three selected oncogenic regulatory axes are HOTAIRM1/miR-182-5p/ERO1A, GOLGA8B/miR-30d-5p/RUNX2, and PDCD6IPP2/miR-23a-3p/HOXA1 (Figure 4F and Table 1). In another part of the screening process, we identified the tumor suppressor regulatory components of miRNAs that interact with down-regulated lncRNAs and down-regulated mRNAs. Using the same rationale, we selected three regulatory axes for tumor suppression; these axes are designated as ANXA2P1/miR-20b-5p/FAM241A (C4orf32), MIR99AHG/miR-218-5p/GPM6A, and SH3RF3-AS1/miR-34a-5p/HECW2 (Figure 4G and Table 1). Th microarray gene expression profiles of lncRNA, mRNA and miRNA validate the expression levels of the selected oncogenic and tumor-suppressive ceRNA modules (Figure 5).

### 3.5. Validation of the Oncogenic and Tumor Suppressor ceRNA Modules in Human Lung Cancer

**Figure 6:**
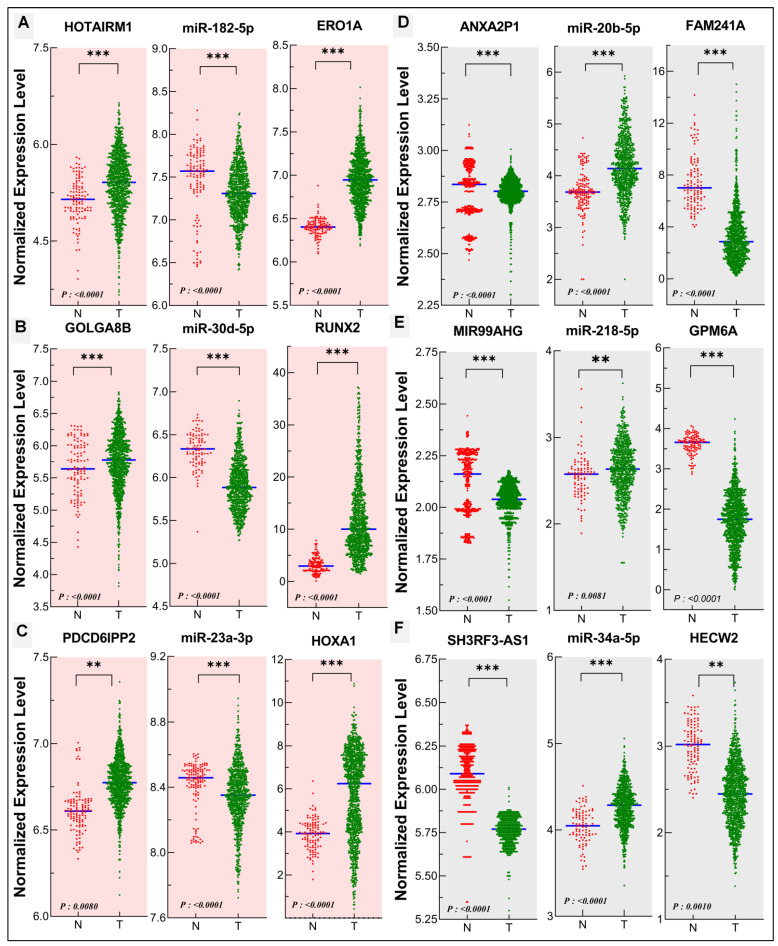
Validation of expression levels of lncRNAs, miRNAs, and mRNAs in identified oncogenic and tumor suppressive ceRNA modules in human lung cancer using gene expression profiling interactive analysis. (**A**–**C**) The oncogenic axes HOTAIRM1/miR-182-5p/ERO1A, GOLGA8B/miR-30d-5p/RUNX2, and PDCD6IPP2/miR-23a-3p/HOXA1. (**D**–**F**) The tumor suppressor axes ANXA2P1/miR-20b-5p/FAM241A, MIR99AHG/miR-218-5p/GPM6A, and SH3RF3-AS1/miR-34a-5p/HECW2. N: normal tissue samples (*n* = 110); T: tumor tissue samples (*n* = 1050) (for tumor suppressive MIR99AHG, SH3RF3-AS1, and ANXA2P1 *n* = 450 and T = 2050). **, *p* ≤ 0.05; ***, *p* ≤ 0.0001.

The lncRNA, miRNA, and mRNA expression data of lung adenocarcinoma (LUAD) and lung squamous cell carcinoma (LUSC) were downloaded, extracted, and combined from multiple TCGA databases and the expression level of each ceRNA element was scattered and plotted in normal and tumor samples. The expression levels of the oncogenic ceRNA module component lncRNAs (HOTAIRM1, GOLGA8B, and PDCD6IPP2) and mRNAs (ERO1A, RUNX2, and HOXA1) identified above were increased, while the expression levels of miR-182-5p, miR-30d-5p, and miR-23a-3p were decreased in tumor tissues compared with normal tissues (Figure 6A–C). In contrast, the expression levels of the tumor suppressive ceRNA module component lncRNAs (ANXA2P1, MIR99AHG, and SH3RF3-AS1) and mRNAs (FAM241A, GPM6A, and HECW2) identified above were reduced while the levels of miR-20b-5p, miR-218-5p, and miR-34a-5p were increased in tumor tissues compared with normal tissues (Figure 6D–F).

### 3.6. Stemness Signature of ceRNA Modules

Physical or regulatory interactions with genes tied directly to stem cell traits may gain stemness signatures. We analyzed whether the identified ceRNA module components are linked to the stemness-related genes.

**Figure 7:**
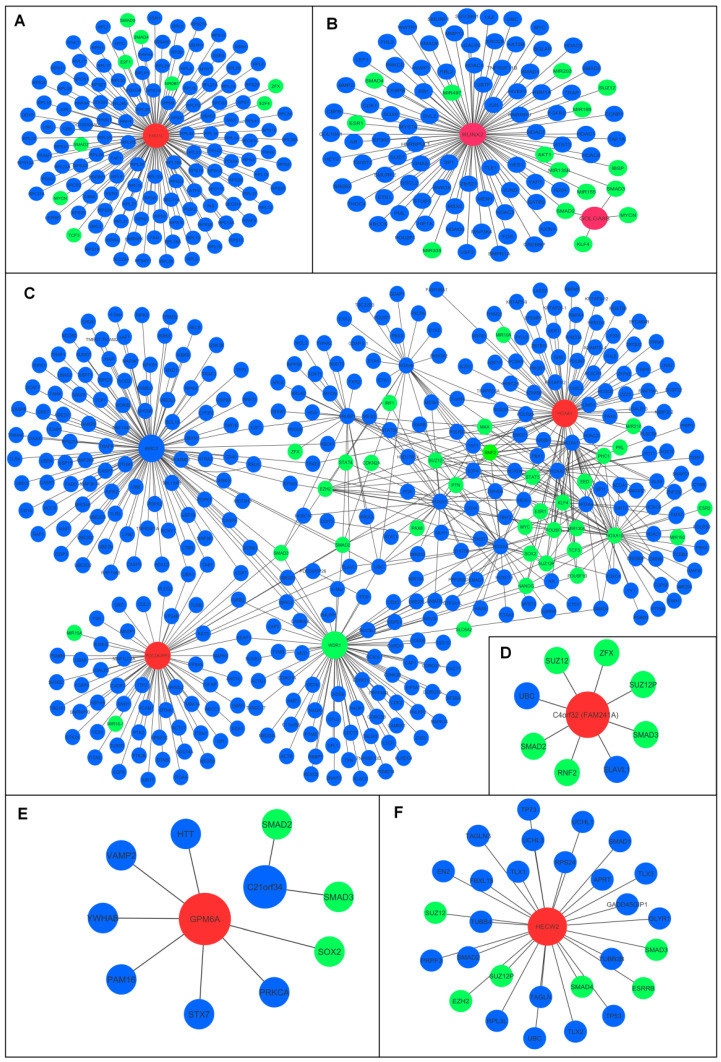
Predicated stemness signatures of selected ceRNA modules. (**A**) ERO1L’s physical and regulatory interactions with stemness-related genes and stemness TFs. (**B**) The interaction of GOLGA8B and RUNX2 partners with the stemness TFs in both the physical and regulatory ways. (**C**) The stemness signature of PDCD6IPP2/miR-23a-3p/HOXA1 module represented via PDCD6IPP2 and HOXA1. (**D**) The stemness signature of ANXA2P1/miR-20b-5p/FAM241A via interaction with stemness-related genes by FAM241A. (**E**) GPM6A interaction with stemness-related genes. (**F**) HECW2 interaction with stemness TFs. The red–green color system represents regulatory interaction, the red–blue color system indicates physical interaction.

It was predicted that the molecular components of the oncogenic HOTAIRM1/miR-182-5p/ERO1A axis possess a significant molecular stemness fingerprinting. ERO1A interacts with nine stemness-related genes (NR0B1, E2F1, E2F4, SMAD2, SMAD3, SMAD4, MYCN, TCF3, and ZFX) in regulatory interaction and has physical interaction with another 128 stemness transcription factor (TF)-related genes. The GOLGA8B/miR-30d-5p/RUNX2 module possesses a significant molecular stemness fingerprinting. GOLGA8B interacts with four stemness-related genes (SMAD2, SMAD3, MYCN, and KLF4), there are 85 stemness-related genes in which RUNX2 is physically related, and an additional 12 stemness-related genes are linked to RUNX2’s regulation (Figure 7A). The interaction between stemness TFs and a key member of the module is the other, and maybe most critical, consideration. GOLGA8B and RUNX2 have regulatory and physical interactions with stemness TFs. RUNX2 and GOLGA8B share a common regulatory interaction with the SMAD2 and SMAD3 stemness-related genes (Figure 7B).

Another oncogenic module, PDCD6IPP2/miR-23a-3p/HOXA1, exhibits a strong molecular fingerprinting of stemness. PDCD6IPP2 and HOXA1 have regulatory connections with 130 stemness-related genes and physical linkages with 428 other genes (Figure 7C), PDCD6IPP2 and HOXA1 are involved in regulating stemness TFs. Physically, HOXA1 binds to 93 TFs, while the remaining 15 interact in a regulatory pattern. Likewise, PDCD6IPP2 binds physically with 36 TFs and contributes to the regulation of an additional four TFs (Figure 7C). The tumor suppressor modules have also contributed to stemness hallmarks via regulatory and physically binding to stemness-related genes and stemness TFs. FAM241A physically binds to stemness-related genes ELAVL1 and UBC. The stemness-related genes SMAD2, SMAD3, RNF2, ZFX, SUZ12, and SUZ12P are regulated by FAM241A (Figure 7D). The module MIR99AHG/miR-218-5p/GPM6A also has a stemness signature through GPM6A’s binding to the stemness-related genes and interaction of GPM6A with stemness TFs (Figure 7E). In addition, the SH3RF3-AS1/miR-34a-5p/HECW2 axis displays a stemness signature via HECW2’s physical binding to 21 stemness TFs and regulatory binding to stemness-related genes SMAD2, SMAD3, SUZ12, SUZ12P, and EZH2 (Figure 7F).

### 3.7. Prognostic Potential Rating of ceRNA Modules in Lung Cancer

Further validation of lung cancer prognosis prediction potential of the identified ceRNA modules was accomplished by analyzing the expression data of nine oncogenic genes (three lncRNAs, three miRNAs, and three mRNAs) and nine tumor suppressor genes (three lncRNAs, three miRNAs, and three mRNAs) via Kaplan–Meier survival analysis to calculate the probability of mortality in lung cancer patients.

**Figure 8:**
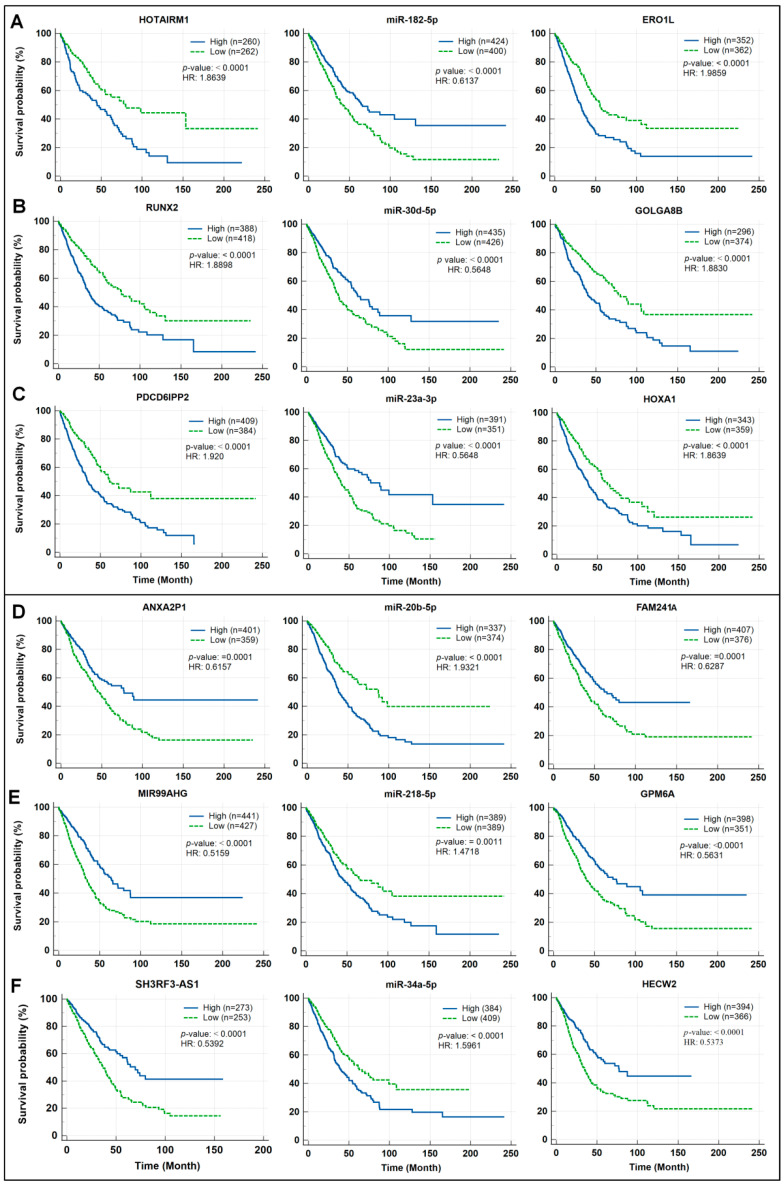
Survival analysis and prognostic performance of selected ceRNA modules in lung cancer. The Kaplan–Meier survival analysis of oncogenic regulatory axes (**A**–**C**) and tumor suppressor regulatory axes (**D**–**F**).

Three oncogenic ceRNA modules were first explored to determine significances of their lung cancer survival rate prediction value. Among the components of the HOTAIRM1/miR-182-5p/ERO1A axis, higher HOTAIRM1 levels exhibit values in predicting worse survival of lung cancer patients. Similar results were obtained with the analysis of ERO1A. In contrast, lower levels of miR-182-5p are associated with a significantly worse lung cancer survival (Figure 8A). Similar results were obtained by analyzing the components from the other two oncogenic ceRNA modules (GOLGA8B/miR-30d-5p/RUNX2 and PDCD6IPP2/miR-23a-3p/HOXA1) (Figure 8B,C).

Next, three tumor suppressive ceRNA modules (ANXA2P1/miR-20b-5p/FAM241A, MIR99AHG/miR-218-5p/GPM6A, and SH3RF3-AS1/miR-34a-5p/HECW2) were analyzed to assess significance of their lung cancer survival rate prediction values. The higher expression levels of three lncRNAs and three mRNAs in the three tumor suppressive ceRNA modules above are associated with significantly better survival of lung cancer patients (Figure 8D–F). In contrast, the lower expression levels of three miRNAs in the three tumor suppressive ceRNA modules are associated with significantly better survival of lung cancer patients (Figure 8D–F).

### 3.8. Diagnostic Performance Assessment of Three ceRNA Modules in Lung Cancer

ROC curve analysis is a well-established method for examining the ability of certain genes/markers to distinguish between disease-prone and disease-free status. In this study, the potential diagnostic values of the three oncogenic and three tumor suppressive ceRNA modules identified above were evaluated using the ROC analysis, the AUC, as well as an estimated score for the diagnostic accuracy, and diagnostic performance indicators (sensitivity and specificity) are used to determine their efficiencies.

**Figure 9:**
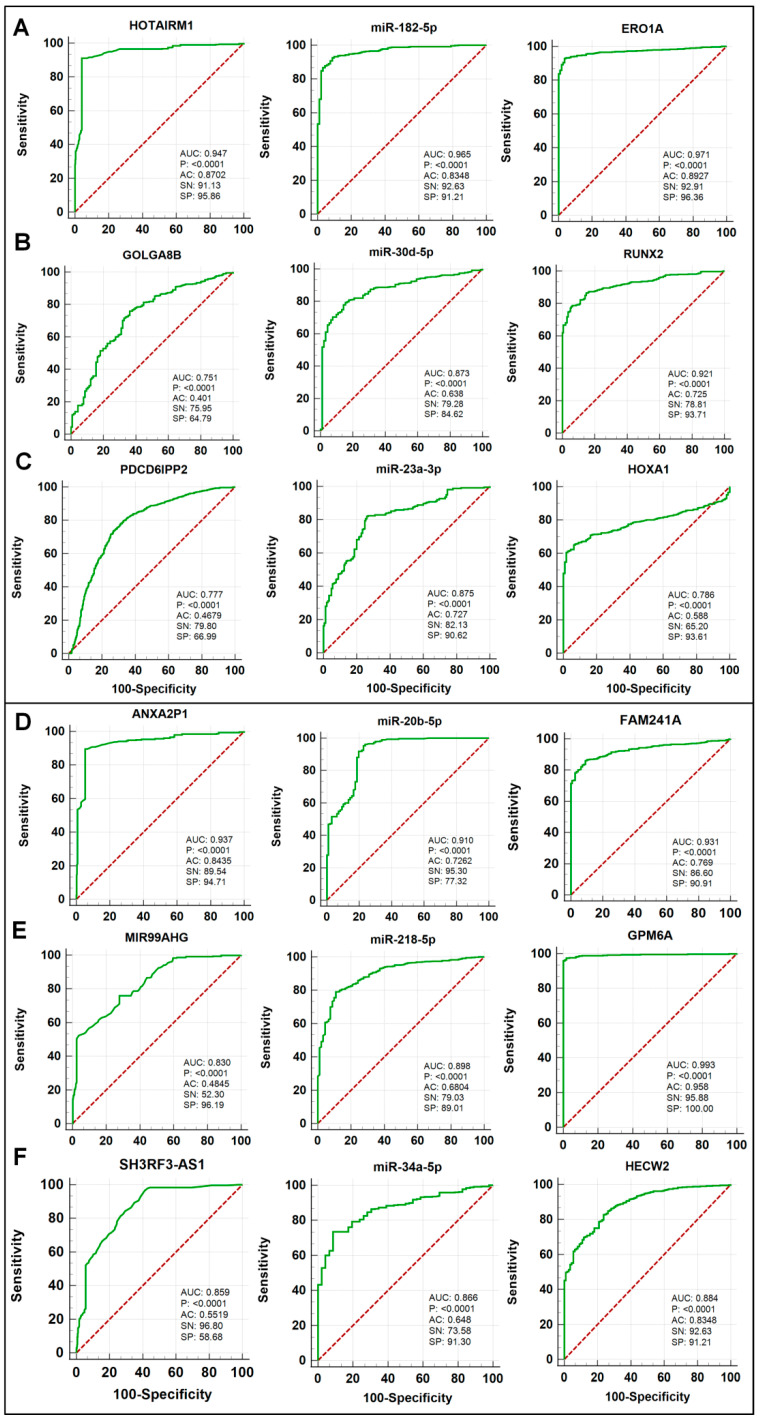
ROC curve and diagnostic performance of ceRNA modules in lung cancer. The ROC curve analysis of oncogenic and tumor suppressor regulatory axes (**A**–**C**) and (**D**–**F**), respectively. AC: accuracy; SN: sensitivity; SP: specificity.

The potential diagnostic values of the components of three oncogenic (HOTAIRM1/miR-182-5p/ERO1A, GOLGA8B/miR-30d-5p/RUNX2, and PDCD6IPP2/miR-23a-3p/HOXA1) and three tumor suppressive (ANXA2P1/miR-20b-5p/FAM241A, MIR99AHG/miR-218-5p/GPM6A, and SH3RF3-AS1/miR-34a-5p/HECW2) ceRNA modules for lung cancer are shown in Figure 9. The *p*-values show that dysregulations of all components of the six ceRNA modules above display significant values for lung cancer diagnosis with AUC values ranging from 0.71 to 0.92, sensitivity (SN) ranging from 54% to 86%, and specificity (SP) ranging from 64% to 100% (Figure 9A–F).

## 4. Discussion

Cr(VI) is a known carcinogen causing lung cancer in humans, however, the mechanism of Cr(VI) lung carcinogenesis has not been well understood. Although lung cancer has been widely studied for decades, the underlying mechanism is still unclear. Lung cancer patients have a 5-year survival rate of 11% after diagnosis [18]. Several studies have found that dysregulations of ncRNAs are closely related to chemical-induced cellular transformation and tumorigenesis [19]. LncRNA and miRNA have been found to have regulatory functions in the process of carcinogenesis via scaffold building of ceRNA [20], by engaging with the 3’ UTR of the target mRNA to regulate the expression of target genes [21]. During carcinogenesis, the regulatory up-lncRNA/down-miRNA/up-mRNA and down-lncRNA/up-miRNA/down-mRNA axes build a three-fold impact on oncogenic and tumor suppressive activities, respectively [22].

In this study, the functional annotation analyses show biological functions such as MF, BP, CC, and biological mechanisms shared by lncRNA, miRNA, and mRNAs in Cr(VI)-transformed cells. Many biological mechanisms including the cell cycle, DNA replication, RNA splicing, and MAPK signaling pathways have significant contributions to oncogenic activities [23], and these mechanisms are shared among oncogenic lncRNAs, miRNAs, and mRNAs. Likewise, in tumor suppressor activities, many biological mechanisms such as TP53 regulation, apoptosomes, and metabolic pathways [24] are shared among lncRNA, miRNA, and mRNA tumor suppressors in Cr(VI)-transformed cells. Many signaling pathways are implicated in cellular transformation and carcinogenesis [25], most notably, there are hundreds of proteins that regulate the processes of initiation of cellular transformation by co-binding with oncogenic or tumor suppressor RNAs [26]. The hallmarks of cancer comprise biological capabilities acquired during the multi-steps of carcinogenesis [27]. The co-binding of proteins to the oncogenic and tumor suppressor lncRNAs, miRNAs, and mRNAs shows highly significant molecular signatures in cancer.

The miRNA–lncRNA and miRNA–mRNA interactions were determined by using miRNA interaction and target prediction algorithms [28]. The predicted miRNAs matched the miRNA array profile from Cr(VI)-transformed cells and the ceRNA network was constructed in oncogenic and tumor suppressor activities. The selected regulatory lncRNA/miRNA/mRNA axis enhances three-fold enrichment in oncogenic or tumor suppressor activities [29]. In this study, the HOTAIRM1/miR-182-5p/ERO1A axis may contribute to lung cancer progression via two collaborators, HOTAIRM1 [30] miR-182-5p [31] and ERO1A [32]. HOTAIRM1 silencing inhibits cell glycolysis metabolism and tumor progression in non-small cell lung cancer [33]. MiR-182-5p overexpression strikingly suppressed oncogenicity of colon cancer cells [34], and ERO1A is a prognostic marker for non-small cell lung cancer [35]. The oncogenic GOLGA8B/miR-30d-5p/RUNX2 axis provides a three-fold impact on lung carcinogenesis.

GOLGA8B knockdown inhibits cell invasion by suppressing the STAT3 signaling pathway in lung squamous cell carcinoma [36]. Down-regulation of miR-30d-5p promotes the proliferation, migration, and invasion of lung squamous cell carcinoma [37]. RUNX2 regulates expression of genes important in tumor cell migration, and has a role in cell proliferation and metastasis of lung cancer [38]. 

The three identified tumor suppressor regulatory axes have a significant influence in restricting the development of lung cancer. The ANXA2P1/miR-20b-5p/FAM241A axis provides a two-fold impact on tumor suppressor activities of cancer progression via ANXA2P1 [39] and miR-20b-5p [40]. In addition, the second tumor suppressor MIR99AHG/miR-218-5p/GPM6A axis provides two-fold lung cancer inhibition via non-coding tumor suppressor gene MIR99AHG in lung adenocarcinoma [41] and miR-218-5p affects lung adenocarcinoma progression [42]. The last tumor suppressor axis, SH3RF3-AS1/miR-34a-5p/HECW2, has a three-fold impact on lung cancer inhibition via SH3RF3-AS1 [43] and miR-34a-5p [44].

The trends of the expression levels of the components in the identified oncogenic and tumor suppressive ceRNA modules in TCGA lung cancer datasets match well with that from the microarray expression profiles of Cr(VI)-transformed cells. These consistencies provide opportunities for analyzing the identified ceRNA modules’ prognostic and diagnostic values in lung cancer [45]. Moreover, the bioinformatics analyses support that the HOTAIRM1/miR-182-5p/ERO1A regulatory axis has significant functions in lung cancer cell proliferation and stemness via regulatory and physical interactions of ERO1A with stemness TFs [46]. miR-182-5p can repress bladder cancer cell migration, invasion, and colony formation [47]. The GOLGA8B/miR-30d-5p/RUNX2 regulatory axis has important functions in lung cancer cell proliferation and stemness via regulatory and physical interactions of GOLGA8B and RUNX2 with both stemness-related genes and stemness TFs [48]. In addition, the oncogenic PDCD6IPP2/miR-23a-3p/HOXA1 axis shows a highly significant stemness signature due to the predicted interaction of HOXA1 with stemness-related genes and stemness TFs at physical and regulatory levels. HOXA1 regulates Nanog and stem cell signaling pathways during early neuro-ectodermal differentiation of embryonic stem cells [49].

The bioinformatics analysis suggests that the tumor suppressor ANXA2P1/miR-20b-5p/FAM241A axis may have an impact on stemness signature via miR-20b-5p. The miR-20b-5p/Oct4 axis is regulated by MALAT1 that mediates stem cell-like properties in human cancer [50]. The MIR99AHG/miR-218-5p/GPM6A axis may display a stemness signature via GPM6A that plays an important role during early events of cellular differentiation and is associated with invasiveness of glioblastoma stem cells [51]. Additionally, the regulatory SH3RF3-AS1/miR-34a-5p/HECW2 axis may modulate stemness traits via the binding of HECW2 with stemness-related genes and stemness TFs. HECW2 regulates cell proliferation and differentiation mediating NEGR1 expression [52].

The prognostic and diagnostic performance scores of ceRNA modules could be detected via overall survival and ROC curve analyses, respectively. The diagnostic and prognostic powers of ceRNA modules could be improved due to their three-fold enrichment [53]. The prognostic performance could be estimated via HR score, the prognostic value is high when HR is greater than 1.00 for up-regulated and lower than 1.00 for down-regulated candidates [54]. Based on HR scores of oncogenic ceRNA modules, they all display significant predictive values for lung cancer patients’ overall survival. Similarly, the HR scores of tumor suppressor ceRNA modules suggest that all three tumor suppressive ceRNA modules also display significant predictive values for lung cancer patients’ overall survival. The outcomes of ROC curve analysis such as AUC value, accuracy, sensitivity, and specificity scores are diagnostic performance indicators, and the one-of-a-kind biomarker has the highest possible value in the diagnostic scores [55]. The previous studies reported that the components in our identified oncogenic and tumor suppressor ceRNA modules may serve as potential biomarkers in many cancer types. For example, the HOTAIRM1 gene has been identified as a potential biomarker for diagnosis of colorectal cancer [56]. ERO1L was found to be a potential marker that can be used to detect lung adenocarcinoma and shapes the immune suppressive tumor microenvironment [57]. GOLGA8B may be utilized as a predictive marker for prostate cancer based on its expression level [58]. The level of miR-30d-5p in blood plasma may serve as a potential biomarker for non-invasive screening of cervical cancer [59]. RUNX2 combined with osteopontin may serve as a new predictive biomarker in resected osteosarcoma [60]. Inhibition of miR-23a-3p promoted osteoblast proliferation and differentiation through targeting the PGC-1α/WNT/β-catenin signaling pathway [61]. HOXA1 is a novel biomarker in the prognosis of head and neck squamous cell carcinoma [62]. It was also found that ANXA2P1 plays an important role as a prognostic biomarker for diffuse gliomas [63]. It was reported that miR-218-5p promotes stemness and cellular regeneration by regulating β-catenin signaling [64]. GPM6A is a potential biomarker and a key regulator in tumorigenesis for rectal tissue [65]. SH3RF3-AS1 is a prognostic marker for predicting survival in hepatocellular carcinoma with cirrhosis [43]. miR-34a-5p may serve as a biomarker for the diagnosis of endometriosis [66]. Together, these findings show that the identified ceRNA modules may not only be critically involved in Cr(VI)-induced cell transformation and tumorigenesis, but may also play important roles in lung cancer in general and have significant prognostic and diagnostic performance values.

## 5. Conclusions

To explore the mechanism of Cr(VI) lung carcinogenesis, we performed lncRNA, mRNA, and miRNA microarray analyses using total RNAs from our previously established Cr(VI)-transformed and passage-matched control human bronchial epithelial cells. The findings from our bioinformatics analyses suggest that the differentially expressed lncRNAs between control and Cr(VI)-transformed cells could be involved in Cr(VI)-induced cell transformation and CSC-like properties by dysregulating RNA–RNA interactions and important biological processes. Indeed, after further analyses of the multi-omics datasets from Cr(VI)-transformed and control cells using the lncRNA–miRNA interaction and miRNA target prediction algorithms, three oncogenic (HOTAIRM1/miR-182-5p/ERO1A, GOLGA8B/miR-30d-5p/RUNX2, and PDCD6IPP2/miR-23a-3p/HOXA1) and three tumor suppressive (ANXA2P1/miR-20b-5p/FAM241A, MIR99AHG/miR-218-5p/GPM6A, and SH3RF3-AS1/miR-34a-5p/HECW2) lncRNA–miRNA–mRNA regulatory axes were identified. Moreover, the findings from analyzing publicly available human lung cancer multi-omics datasets reveal that the identified three oncogenic and three tumor suppressive lncRNA–miRNA–mRNA regulatory axes in Cr(VI)-transformed cells have significant diagnostic and prognostic prediction values in human lung cancer. Collectively, the findings from this study suggest that dysregulations of lncRNAs and RNA–RNA interactions and the three oncogenic and three tumor suppressive lncRNA–miRNA–mRNA regulatory axes identified may play important roles in Cr(VI) carcinogenesis and lung cancer in general as well.

## Figures and Tables

**Table 1:** The selected potential oncogenic and tumor suppressor ceRNA modules (lncRNA, miRNA, and mRNA).

	lncRNA	miRNA	mRNA
**Oncogenic ceRNA modules**			
**A**	HOTAIRM1	miR-182-5p	ERO1A
**B**	GOLGA8B	miR-30d-5p	RUNX2
**C**	PDCD6IPP2	miR-23a-3p	HOXA1
**Tumor suppressor ceRNA modules**			
**D**	ANXA2P1	miR-20b-5p	FAM241A (C4orf32)
**E**	MIR99AHG	miR-218-5p	GPM6A
**F**	SH3RF3-AS1	miR-34a-5p	HECW2

## Data Availability

Data supporting reported results is included in the Supplementary Materials.

## Supplementary Materials

The following supporting information can be downloaded at: https://www.mdpi.com/article/10.3390/biomedicines10102334/s1; Supplementary Excel File S1: DE_lncRNAs_BEAS-2B Cr(VI) Microarray Profile; Supplementary Excel File S2: DE_miRNAs_BEAS-2B Cr(VI) Microarray Profile; Supplementary Excel File S3: DE_mRNAs_BEAS-2B Cr(VI) Microarray Profile; Supplementary Excel File S4: Biological Mechanisms and GO of lncRNA Profile; Supplementary Excel File S5: Biological Mechanisms and GO of miRNA Profile; Supplementary Excel File S6: Biological Mechanisms and GO of mRNA Profile; Supplementary Excel File S7: PP interaction Network (PP interaction with DEGs of lncRNA and mRNA); Supplementary Excel File S8: PP interaction Network (mRNA interaction with DE miRNA); Supplementary Excel File S9: Stemness Signature of Selected ceRNA modules; Supplementary Table S1: The significant gene ontology terms (MF, BP and CC) of DE lncRNAs between BEAS-2B-Cr (VI) and BEAS-2B; Supplementary Table S2: List of gene ontology keywords the (MF, BP and CC) of the DE miRNAs between BEAS-2B-Cr (VI) and BEAS-2B; Supplementary Table S3: List of gene ontology keywords the (MF, BP and CC) of the DE mRNAs between BEAS-2B-Cr (VI) and BEAS-2B.
