# Supplementary material for: The Oncogenic and Tumor Suppressive Long Non-Coding RNA–microRNA–Messenger RNA Regulatory Axes Identified by Analyzing Multiple Platform Omics Data from Cr(VI)-Transformed Cells and Their Implications in Lung Cancer
Source: Biomedicines. 2022 Sep 20;10(10):2334. doi: 10.3390/biomedicines10102334 (PMC9598927; doi:10.3390/biomedicines10102334)
Supplement: Supplementary file 1 [file biomedicines-10-02334-s001.zip › supplementary Tables 1-3.pdf]

Supplementary data

Table S1. The signficant gene ontology terms (MF, BP and CC) of DE lncRNAs between BEAS-2B-Cr (VI) and BEAS-2B.

Source	Term name	Term ID	P value	ES	size
GO:MF	DNA binding	GO:0003677	0.00055784	3.25349268	8
GO:MF	nucleic acid binding	GO:0003676	0.00046400	3.33348389	15
GO:MF	catalytic activity	GO:0003824	0.00039410	3.40438916	24
GO:MF	heterocyclic compound binding	GO:1901363	0.00031208	3.50573022	22
GO:MF	adenyl nucleotide binding	GO:0030554	0.00021264	3.67236525	8
GO:MF	binding	GO:0005488	0.00015901	3.79858485	49
GO:MF	ion binding	GO:0043167	0.00007596	4.11942865	18
GO:MF	transcription cis-regulatory region binding	GO:0000976	0.00006833	4.16540765	6
GO:MF	RNA binding	GO:0003723	0.00003405	4.46783891	9
GO:MF	molecular function regulator	GO:0098772	0.00001965	4.70654419	7
GO:CC	endomembrane system	GO:0012505	0.00040385	3.39378099	10
GO:CC	organelle	GO:0043226	0.00032433	3.48901288	55
GO:CC	intracellular organelle	GO:0043229	0.00016127	3.79244641	52
GO:CC	membrane-bounded organelle	GO:0043227	0.00008153	4.08868256	53
GO:CC	protein-containing complex	GO:0032991	0.00006595	4.18078520	18
GO:CC	cytosol	GO:0005829	0.00005734	4.24157791	14
GO:CC	nucleolus	GO:0005730	0.00003527	4.45261067	10
GO:CC	membrane	GO:0016020	0.00000999	5.00043016	29
GO:CC	intracellular anatomical structure	GO:0005622	0.00000647	5.18918299	59
GO:CC	Mitochondrion	GO:0005739	0.00000562	5.25047084	12
GO:BP	gene silencing by miRNA	GO:0035195	0.00082850	3.08170882	22
GO:BP	cellular component organization	GO:0016043	0.00009241	4.03426397	22
GO:BP	RNA metabolic process	GO:0016070	0.00004858	4.31358719	20
GO:BP	negative regulation of gene expression	GO:0010629	0.00003478	4.45868773	27
GO:BP	localization	GO:0051179	0.00003401	4.46838042	16
GO:BP	signaling	GO:0023052	0.00000501	5.30059592	22
GO:BP	negative regulation of metabolic process	GO:0010605	0.00000474	5.32455438	10
GO:BP	cell cycle	GO:0007049	0.00000095	6.02045163	6
GO:BP	metabolic process	GO:0008152	0.00000089	6.05119107	65
GO:BP	cellular process	GO:0009987	0.00000023	6.64662143	69

Table S2. List of gene ontology keywords the (MF, BP and CC) of the DE miRNAs between BEAS-2B-Cr (VII) and BEAS-2B.

Source	Term name	Term ID	P value	ES	size
GO:MF	cation binding	GO: 0043169	0.02538840	1.59536467	29
GO:MF	copper-dependent protein binding	GO: 0032767	0.00150380	2.82280992	2
GO:MF	GTPase activating protein binding	GO: 0032794	0.00071500	3.14569396	9
GO:MF	molecular function	GO: 0003674	0.00060700	3.21681131	75
GO:MF	protein kinase inhibitor activity	GO: 0004860	0.00041000	3.38721614	9
GO:MF	enzyme binding	GO: 0019899	0.00014100	3.85078089	100
GO:MF	kinase inhibitor activity	GO: 0019210	0.00012300	3.91009489	10
GO:MF	ubiquitin protein ligase binding	GO: 0031625	0.00012100	3.91721463	50
GO:MF	protein binding	GO: 0005515	0.00000253	5.59687948	121
GO:MF	RNA binding	GO: 0035198	0.00000203	5.69250396	87
GO:CC	nucleoplasm	GO: 0005654	0.00200150	2.69864441	162
GO:CC	protein-DNA complex	GO: 0032993	0.00193170	2.71406032	18
GO:CC	extracellular exosome	GO: 0070062	0.00120300	2.91973437	41
GO:CC	cortical actin cytoskeleton	GO: 0030866	0.00073400	3.13430394	9
GO:CC	ribonucleoprotein complex	GO: 1990904	0.00028000	3.55284197	40
GO:CC	nucleus	GO: 0005634	0.00022200	3.65364703	189
GO:CC	melanosome	GO: 0042470	0.00021500	3.66756154	18
GO:CC	cytoplasm	GO: 0005737	0.00008440	4.07365755	88
GO:CC	secretory granule	GO: 0030141	0.00008010	4.09636748	7
GO:CC	cytosol	GO: 0005829	0.00001370	4.86327943	98
GO:BP	cellular response to hypoxia	GO: 0071456	0.00015100	3.82102305	43
GO:BP	cellular response to tumor necrosis factor	GO: 0071356	0.00014300	3.84466396	13
GO:BP	regulation of cell death	GO: 0010941	0.00013400	3.87289520	80
GO:BP	cellular macromolecule biosynthetic process	GO: 0034645	0.00013200	3.87942607	147
GO:BP	response to ionizing radiation	GO: 0010212	0.00012500	3.90308999	20
GO:BP	regulation of protein kinase activity	GO: 0045859	0.00007280	4.13786862	60
GO:BP	regulation of sprouting angiogenesis	GO: 1903670	0.00001940	4.71219827	25
GO:BP	organelle organization	GO: 0006996	0.00000596	5.22475374	82
GO:BP	cellular process	GO: 0009987	0.00000068	6.16877031	86
GO:BP	biological process	GO: 0008150	0.00000066	6.18375870	60

Table S3. List of gene ontology keywords the (MF, BP and CC) of the DE mRNAs between BEAS-2B-Cr (VII) and BEAS-2B.

Source	Term name	Term ID	P value	ES	size
GO:MF	small molecule binding	GO:0036094	0.00000000	184.60601	636
GO:MF	metal ion binding	GO:0046872	0.00000000	233.25730	939
GO:MF	cation binding	GO:0043169	0.00000000	236.50131	955
GO:MF	nucleic acid binding	GO:0003676	5e-324	312.48822	1052
GO:MF	catalytic activity	GO:0003824	5e-324	323.30622	1386
GO:MF	ion binding	GO:0043167	5e-324	323.30622	1409
GO:MF	heterocyclic compound binding	GO:1901363	5e-324	323.30622	1515
GO:MF	organic cyclic compound binding	GO:0097159	5e-324	323.30622	1536
GO:MF	protein binding	GO:0005515	5e-324	323.30622	3398
GO:MF	binding	GO:0005488	5e-324	323.30622	3770
GO:CC	nucleoplasm	GO: 0005654	5e-324	323.30622	1460
GO:CC	protein-DNA complex	GO: 0032993	5e-324	323.30622	1903
GO:CC	extracellular exosome	GO: 0070062	5e-324	323.30622	2020
GO:CC	cortical actin cytoskeleton	GO: 0030866	5e-324	323.30622	2878
GO:CC	ribonucleoprotein complex	GO: 1990904	5e-324	323.30622	2878
GO:CC	nucleus	GO: 0005634	5e-324	323.30622	3102
GO:CC	melanosome	GO: 0042470	5e-324	323.30622	3131
GO:CC	cytoplasm	GO: 0005737	5e-324	323.30622	3285
GO:CC	secretory granule	GO: 0030141	5e-324	323.30622	3485
GO:CC	cytosol	GO: 0005829	5e-324	323.30622	4005
GO:BP	macromolecule metabolic process	GO:0043170	5e-324	323.30622	2296
GO:BP	nitrogen compound metabolic process	GO:0006807	5e-324	323.30622	2428
GO:BP	regulation of cellular process	GO:0050794	5e-324	323.30622	2492
GO:BP	primary metabolic process	GO:0044238	5e-324	323.30622	2535
GO:BP	cellular metabolic process	GO:0044237	5e-324	323.30622	2602
GO:BP	regulation of biological process	GO:0050789	5e-324	323.30622	2609
GO:BP	organic substance metabolic process	GO:0071704	5e-324	323.30622	2644
GO:BP	biological regulation	GO:0065007	5e-324	323.30622	2755
GO:BP	metabolic process	GO:0008152	5e-324	323.30622	2769
GO:BP	cellular process	GO:0009987	5e-324	323.30622	3741
